# *Caenorhabditis elegans* SMA-10/LRIG Is a Conserved Transmembrane Protein that Enhances Bone Morphogenetic Protein Signaling

**DOI:** 10.1371/journal.pgen.1000963

**Published:** 2010-05-20

**Authors:** Tina L. Gumienny, Lesley MacNeil, Cole M. Zimmerman, Huang Wang, Lena Chin, Jeffrey L. Wrana, Richard W. Padgett

**Affiliations:** 1 Waksman Institute, Department of Molecular Biology and Biochemistry, Cancer Institute of New Jersey, Rutgers University, Piscataway, New Jersey, United States of America; 2 Program in Molecular Biology and Cancer, Samuel Lunenfeld Research Institute, Mount Sinai Hospital, Toronto, Canada; 3 Department of Molecular and Medical Genetics, University of Toronto, Toronto, Canada; University of California San Diego, United States of America

## Abstract

Bone morphogenetic protein (BMP) pathways control an array of developmental and homeostatic events, and must themselves be exquisitely controlled. Here, we identify *Caenorhabditis elegans* SMA-10 as a positive extracellular regulator of BMP–like receptor signaling. SMA-10 acts genetically in a BMP–like (Sma/Mab) pathway between the ligand DBL-1 and its receptors SMA-6 and DAF-4. We cloned *sma-10* and show that it has fifteen leucine-rich repeats and three immunoglobulin-like domains, hallmarks of an LRIG subfamily of transmembrane proteins. SMA-10 is required in the hypodermis, where the core Sma/Mab signaling components function. We demonstrate functional conservation of LRIGs by rescuing *sma-10(lf)* animals with the *Drosophila* ortholog *lambik*, showing that SMA-10 physically binds the DBL-1 receptors SMA-6 and DAF-4 and enhances signaling *in vitro*. This interaction is evolutionarily conserved, evidenced by LRIG1 binding to vertebrate receptors. We propose a new role for LRIG family members: the positive regulation of BMP signaling by binding both Type I and Type II receptors.

## Author Summary

Bone morphogenetic protein (BMP) family members, small secreted signaling molecules, play diverse roles in development and homeostasis. Uncontrolled BMP signaling results in a variety of disorders and diseases. BMPs signal to receiving cells through two receptor types, which act together to propagate the BMP signal within cells. To understand how BMP signaling is controlled, we used the nematode *Caenorhabditis elegans* to identify conserved regulators of BMP signaling. Here, we characterize SMA-10, the first extracellular positive regulator of DBL-1/BMP receptor-mediated signaling. SMA-10 is a new member of a family with leucine rich repeats and immunoglobulin-like domains (LRIG). SMA-10 physically binds the two types of DBL-1/BMP receptor. We demonstrate conservation of LRIG function by showing that a *Drosophila melanogaster* LRIG can functionally substitute for loss of *C. elegans* SMA-10/LRIG, that *C. elegans* SMA-10 can directly promote mammalian BMP signaling in cells, and that mammalian LRIG1 interacts with BMP receptors. Our work establishes a role for LRIGs in BMP regulation through binding both types of BMP receptor.

## Introduction

Bone morphogenetic protein (BMP) receptor serine/threonine kinases (BMPRs) are pivotal signal transducers for the small, secreted BMP morphogens, members of the transforming growth factor β (TGF- β) superfamily (comprising subfamilies of TGF- βs, BMPs, activins, and others) [1], [2]. BMP dimers released from neighboring cells are received by these receptors, which leads to an intracellular cascade of transcriptional events. Depending on the specific pathway, cell type and milieu, these events result in a diverse array of cellular processes, from dorsal-ventral specification to cell cycle control and programmed cell death [3]. Understanding how growth factor pathways are regulated may lead not only to a better understanding of their normal physiological roles, but may also lead to potential treatments for a wide range of disorders and diseases [4], [5].

Secreted BMP dimers travel through the extracellular matrix to activate their receptors. Originally thought to be a process of simple diffusion, the movement of TGF-β superfamily members is now recognized to be highly regulated [6]. Many factors play a role in facilitating or preventing BMP ligand access to receptor. Post-translational processing and proteolysis of ligand, as well as seclusion of ligand by extracellular matrix (ECM) components like integrins and proteoglycans, for example, determine whether a ligand dimer can interact with its receptors [7]. Not only is the BMP's progress exquisitely controlled, but the receptors themselves are also subject to regulation [6], [8]. Inside the cell, receptor phosphorylation is inhibited by phosphatases, and SARA and Smurf proteins target receptors for polyubiquitination and degradation [8]. Outside the cell, the receptor complex can be inactivated by the decoy type I receptor BAMBI [9]. Coreceptors betaglycan/TGFβ receptor II (TGFβR3) and endoglin can bind certain BMPS and deliver them to receptors [10]–[14]. Endoglin also associates with select type I and type II receptors [13].

Pioneer studies in *Caenorhabditis elegans* and *Drosophila melanogaster* have identified components of the pathway and furthered understanding of BMP signaling [15], [16]. These studies have identified the conserved core of the signaling pathway, including the ligand, the type I and type II receptors, and the Smads. In *C. elegans*, a BMP-like pathway controls body size and male tail development (the Sma/Mab pathway). The receptors for the ligand DBL-1 are SMA-6 (type I) and DAF-4 (type II) [17], [18]. Receptor signals are transduced through the Smads SMA-2, SMA-3, and SMA-4 [19]. As in mammals, co-transcription factors that act with the Smads have been discovered, including SMA-9/Schnurri and RNT-1/RUNX [20], [21]. The body size phenotype of the Sma/Mab pathway is very sensitive to dose, indicating that the core pathway is tightly controlled [17], [18], [22], [23]. The only identified extracellular regulator of this pathway has been LON-2, a conserved heparan sulfate proteoglycan that binds DBL-1/BMP and attenuates pathway signaling [24].

To identify novel components of BMP signaling, we performed traditional forward genetic screens for mutations affecting the Sma/Mab signaling pathway ([25], unpublished results). From these genetic screens, we identified, cloned, and characterized *sma-10*. SMA-10 is a positive regulator of Sma/Mab signaling, and is absolutely and specifically required for body size regulation. It is a member of a cell surface localized family of proteins with leucine rich repeats and immunoglobulin-like domains (LRIG). The function of SMA-10 in BMP pathway signaling is conserved, as the *Drosophila* ortholog *lambik* rescues the body size defect of *sma-10(lf)* animals. Furthermore, SMA-10 promotes BMP signaling in mammalian cells. SMA-10 binds the pathway receptors SMA-6 and DAF-4 but not the BMP DBL-1, and a mammalian ortholog, LRIG1 (leucine-rich and immunoglobulin-like domains-1), also binds both type I and type II receptors. These studies identify a uniquely acting positive regulator of BMP signaling, SMA-10/LRIG, that directly interacts with type I and type II receptors from *C. elegans* to mammals.

## Results

### *sma-10(lf)* Alleles Were Identified in Screens for Small Body Size

The first small *C. elegans* mutants were identified in a large-scale screen for morphology and mobility mutants [26]. Their role in BMP signaling was elucidated when *sma-2*, *sma-3* and *sma-4* were characterized [19]. In an effort to identify additional genes that act in BMP signaling, we performed two genetic screens. From the first screen, in which body size mutant F_2_ animals were isolated from mutagenized N2/wild type P_0_ animals, two *sma-10* alleles, *wk26* and *wk66*, were identified [25]. From a *lon-2(e678)* suppressor screen, three additional alleles, *wk88*, *wk89*, and *wk90*, were identified and confirmed by complementation and sequencing.

### *sma-10* Mutants Display Body Size Defects But No Male Tail Abnormalities

**Figure 1:**
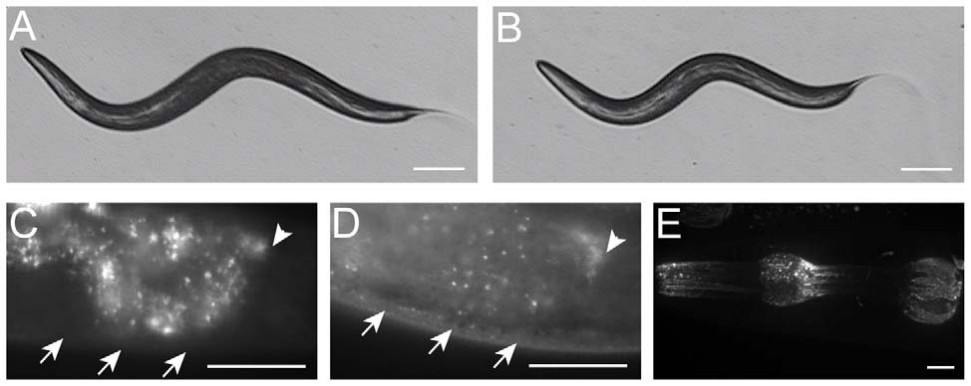
*sma-10* is required for normal body size. Microscope images of (A) a wild-type (N2) hermaphrodite and (B) a *sma-10(wk66)* hermaphrodite, both taken at 24 hours after the L4/adult molt (same magnification on a dissecting microscope, scale bar represents 0.1 mm). SMA-10:GFP is expressed in hypodermis (D) and pharynx muscle (E). No hypodermal fluorescence was detected in non-transgenic animals (C). Weak expression was detected in hypodermis (20-fold longer exposure of image in (C) than (D)). Arrows point to hypodermis in (C,D). Arrowheads point to the end of the intestine in (C,D). Intestinal autofluorescence is visible to the left of the arrowheads in (C,D). Strong expression of the SMA-10:GFP full-length fusion construct was observed in the pharynx muscle. Expression of the *ttx-3::rfp* marker in the AIY neuron is also visible in the upper right corner (E). Scale bars in (C–E) represent 0.01 mm. Anterior is to the left in all panels.

The Sma/Mab pathway regulates both body size and male tail development. A reduction of *dbl-1* pathway activity results in animals that are 55%–85% wild-type length [22],[23]. *sma-10(lf)* animals share the small body size defect, ranging from 79% to 88% the length of wild-type animals (Figure 1A and 1B, Table 1). The *dbl-1* pathway also regulates the development and patterning of male tail structures [17]–[19], [22], [23], with mating spicules and sensory rays 5–7 being primarily affected. We therefore asked if *sma-10* is also involved in patterning the male tail. Our studies revealed that all five alleles of *sma-10*, including a presumed null *(wk88)*, have wild-type male tail rays and spicules (data not shown). These data suggest that SMA-10 is specifically involved in BMP signaling to control body size but not male tail development or patterning.

**Table 1:** Quantitation of *sma-10* mutant body size.

genotype	p value	% WT	n
wild type		100±1	46
*sma-10(wk26)*	<0.001	88±2	46
*sma-10(wk66)*	<0.001	81±2	41
*sma-10(wk88)*	<0.001	79±2	42
*sma-10(wk89)*	<0.001	82±2	26
*sma-10(wk90)*	<0.001	83±2	37

Body perimeters of staged one-day adult hermaphrodites were measured. The p value is the probability that the null hypothesis, that the mean body length of each *sma-10* mutant line is the same as the wild-type mean body length, is true. “% WT” is the ratio of the indicated strain mean to the wild-type strain mean ±95% confidence interval. n, number of animals measured.

### SMA-10 Acts in the Sma/Mab Pathway

Because small body size is a hallmark phenotype of mutants in the Sma/Mab pathway, we asked if *sma-10* genetically interacts with Sma/Mab pathway members. To determine the functional order of SMA-10 relative to Sma/Mab pathway members, we performed epistasis tests. We generated animals with mutations in *sma-10* and DBL-1/BMP pathway genes that alone give opposite phenotypes (small and long, respectively), and asked what the terminal phenotype was for these strains (small or long). In a regulatory pathway, the terminal phenotype is a result of the loss of gene product with the most downstream effect bypassing the requirement for the second, upstream gene product. We created double mutant animals of *sma-10* with *lon-2*, overexpressed *dbl-1*, or over expressed *sma-6*. We found that the small body size phenotype of *sma-10(lf)* was the terminal phenotype in double mutant animals with either *lon-2* or over expressed *dbl-1*, but not for overexpressed *sma-6* (Table 2). These results suggest that SMA-10 functions in the unique position between the receptor SMA-6 and the ligand DBL-1.

**Table 2:** *sma-10* acts between ligand *dbl-1* and receptor *sma-6*.

genotype	p value	% WT	n
wild type	-	100±3	33
*sma-10(wk66)*	<0.001	84±3	31
*sma-10(wk89)*	<0.001	82±2	26
*lon-2(e678)*	<0.001	114±2	29
*sma-10(wk89); lon-2(e678)*	0.035 a	79±2	23
*ctIs40 (dbl-1(+))*	<0.001	125±4	17
*sma-10(wk66); ctIs40 (dbl-1(+))*	>0.5 b	84±2	30
*sma-6(wk7)*	<0.001	77±3	31
*sma-6(wk7); texEx190 (sma-6(+))*	<0.001 c	88±3	31
*sma-10(wk66); texEx190 (sma-6(+))*	<0.001 b	99±5	12

**^a^**Compared to *sma-10(wk89)*.

**^b^**Compared to *sma-10(wk66)*.

**^c^**Compared to *sma-6(wk7)*.

*ctIs40* is an integrated array that overexpresses a cosmid fragment containing wild-type *dbl-1* promoter and gene. *texEx190* is an extrachromosomal array that drives expression of a functional SMA-6:GFP fusion protein from the *sma-6* promoter. The p value is the probability that the null hypothesis, that the mean body length of each mutant line is the same as the background body length, is true. “% WT” is the ratio of the indicated strain mean to the wild-type strain mean ±95% confidence interval. n, number of animals measured.

### *sma-10* Encodes a Protein with Leucine-Rich Repeats and Immunoglobulin-Like Domains

**Figure 2:**
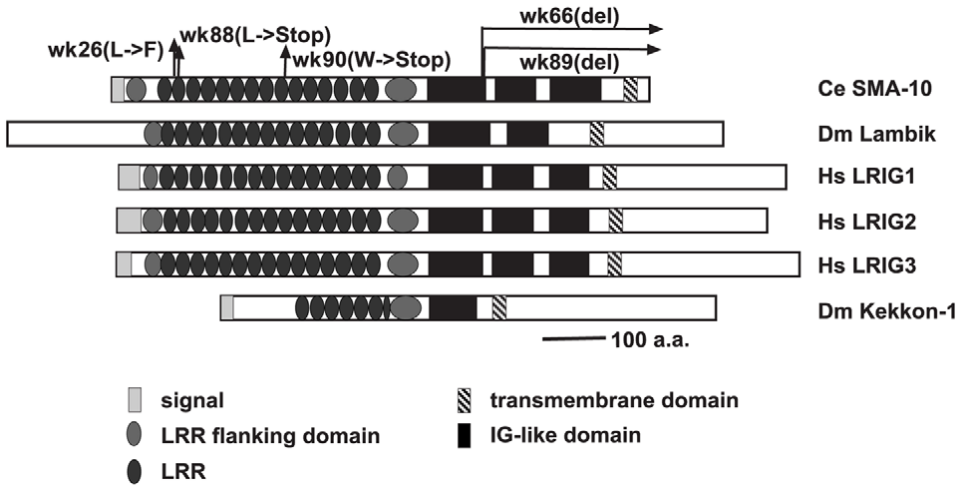
Structural comparison of SMA-10 to *Drosophila* Lambik and mammalian LRIGs. Schematic diagram of *C. elegans* SMA-10, *Drosphila* Lambik, human LRIG1, LRIG2, and LRIG3 and *Drosophila* Kekkon 1. Domains/motifs are represented as follows: N-terminal signal sequences, light gray rectangles; leucine rich repeat N- and C-terminal domains, medium gray ovals; leucine-rich repeats, dark gray ovals; immunoglobulin-like domains, black boxes; and transmembrane domains, striped rectangles. Arrows above the *C. elegans* protein sequence indicate mutations affecting the SMA-10 protein. Domains were placed according to SMART analyses [62], Guo et al (LRIGs) [27], and MacLaren et al (Kekkon 1) [63].

To understand the molecular nature of *sma-10*, we mapped and cloned the gene. *sma-10* is located at LG IV: −26.82. Cosmid T21D12 conferred rescue of the *sma-10(wk66)* small phenotype, as does a *sma-10* cDNA (Table S1). *sma-10* encodes an 881 amino acid protein of the LRIG family (leucine rich repeats and immunoglobulin-like domains). SMA-10 is composed of an N-terminal signal sequence (amino acids 1–20), fifteen leucine-rich repeats (LRRs) flanked by an LRR N-terminal domain at amino acids 24–56 and an LRR C-terminal domain ending at amino acid 499, three immunoglobulin domains (spanning amino acids 503 to 802), a transmembrane domain (amino acids 839 to 861), and a short (19 amino acid) intracellular domain from amino acids 862 to 881 (Figure 2). The C-terminal tail is not conserved between *C. elegans*, *Drosophila* and mammals. This protein structure, which is largely extracellular and is transmembrane-bound, is consistent with the order of gene function, which places SMA-10 between the secreted DBL-1/BMP and its membrane-bound receptors.

Sequencing DNA of mutant *sma-10* strains confirmed lesions within T21D12.9 (Figure 2). *sma-10(wk26)* contains a T to C bp change at position 572, a substitution that changes a conserved leucine to a phenylalanine in the second LRR at amino acid 102. A deletion of 251 bps generates *sma-10(wk66)* (on cosmid T21D12 from base pairs 21092 to 21342, beginning in the gene at the end of exon 9). This transcript is predicted to result in a truncated protein with 609 a.a. of SMA-10 and 10 a.a. of novel sequence before terminating, deleting sequences after the first immunoglobulin-like domain. *wk88* introduces a stop codon into amino acid position 112 in the LRR2, resulting in a severely truncated protein, and is a presumed null allele. *wk89* is a 975 bp deletion from position 21301 to 22275 in T21D12, starting in intron 9 and ending in intron 11, deleting the protein after amino acid 611 and creating a frame shift and premature termination sequence, removing sequences after the first immunoglobulin-like domain. *wk90* changes sequence encoding Trp 286 to a stop codon, deleting sequences after LRR9.

Multiple splice variants are predicted by cDNA sequencing (Wormbase.org). yk352c5, a full-length 2.6 kb cDNA of the longest splice variant T21D12.9a, driven by 1.2 kb of upstream *sma-10* promoter sequence, is sufficient to confer rescue of *sma-10(wk66)* animals (Table S1). A function for the shorter splice variants (T21D12.9b, T21D12.9c.1, and T21D12.9c.2) is not known.

Database searches reveal orthologs in other metazoans. The hallmark of this family is the presence of fifteen leucine-rich repeats (LRRs) and three immunoglobulin-like repeats (Ig-like) followed by a transmembrane domain. In *Drosophila*, there is one SMA-10 ortholog, Lambik (www.flybase.org), and in vertebrates, there are three known *sma-10* orthologs, LRIG1, LRIG2, and LRIG3 [27]. *lambik* corresponds to CG8434, but no phenotypic characterization has been published. A distantly related, distinct family is composed of the insect-specific Kekkon members, which have six LRRs and one Ig–like domain. One of these, Kekkon 1 (kek1), plays a role in inhibiting the epidermal growth factor receptor (EGFR) [28]. Another Kekkon, Kekkon5 (kek5), interacts genetically with a BMP signaling pathway [29].

### *sma-10* Is Required in the Hypodermis to Regulate Body Size

Although core Sma/Mab pathway components are expressed in many tissues, they are all required in the hypodermis for body size regulation. To determine if SMA-10 is also needed in these cells, we first asked where *sma-10* is expressed. We created a functional translational fusion of SMA-10 with GFP at the C-terminus and expressed it using the *sma-10* promoter. Expression of SMA-10:GFP was visible, though faint, in the hypodermis (Figure 1C and 1D), consistent with SMA-10's genetically identified role as a regulator of the DBL-1 signaling pathway, which functions in the hypodermis to regulate body size. Expression in the hypodermis of other DBL-1 pathway genes is also low [24], [30]–[33].

Bright fluorescent expression was also observed in the pharynx of animals from embryos to adults (Figure 1E). Pharyngeal expression was localized to the muscle segments pm1 (anterior cell of the procorpus), pm4 (metacarpus/anterior bulb), the anterior part of pm5 (isthmus), and pm7 (terminal bulb) (Figure 1E). Expression of SMA-10:GFP localized to both the cell surface and to puncta within the cells (Figure 1E). Expression of mRNA corresponding to the *sma-10* locus has also been reported in intestine and renal gland cells in both larvae and adult animals [34]. Digestive tract expression (in the pharynx and intestine) of other Sma/Mab pathway members has been reported [18], [32], [33], [35], [36], and may reflect a proposed role for this pathway in an innate immune response [37], [38].

We then asked where SMA-10 is required for body size regulation. Because of its expression pattern and previous experiments showing that DBL-1/BMP pathway components are required in the hypodermis, we drove the expression of full-length genomic *sma-10* from pharyngeal- or hypodermal-specific promoters in *sma-10(wk66)* animals and assayed for rescue of body size. We found that expression of *sma-10(+)* in hypodermis (using the *rol-6* promoter), but not pharynx (using the *myo-2* promoter), was sufficient to rescue animals to wild-type length (Table 3).

**Table 3:** Hypodermal *sma-10* expression rescues the body size defect of *sma-10(lf)* animals.

Genotype	p value	% WT	n
wild type	-	100±2	35
*sma-10(wk66)*	<0.001	84±3	31
*sma-10(wk66);wkEx91 (myo-2p::sma-10(+))* pharynx	>0.5 a	84±3	11
*sma-10(wk66);wkEx92 (rol-6p::sma-10(+))* hypodermis	<0.001 a	98±3	38

**^a^**compared to *sma-10(wk66)*.

The *myo-2* promoter drives pharyngeal expression, and the *rol-6* promoter drives hypodermal expression. The p value is the probability that the null hypothesis, that the mean body length of each mutant line is the same as the background body length, is true. “% WT” is the ratio of the indicated strain mean to the wild-type strain mean ±95% confidence interval. n, number of animals measured.

### *Drosophila* LRIG *lambik* Rescues *sma-10(lf)* Animals

Lambik is a *Drosophila* LRIG that is orthologous to vertebrate LRIGs and to SMA-10. Although the function of Lambik is currently unknown, we asked whether *lambik* could functionally substitute for *sma-10* in *C. elegans*. We drove *Drosophila lambik* cDNA from the *sma-10* promoter in *sma-10(wk66)* animals. Transgenic animals were rescued to the wild-type body size (Table 4), thereby showing functional conservation between divergent *Drosophila* and *C. elegans* LRIGS.

**Table 4:** *Drosophila lambik* rescues the body size defect of *sma-10(lf)* animals.

Genotype	p value	% WT	n
wild type	-	100±2	39
*sma-10(wk66)*	<0.001	84±3	31
*sma-10(wk66); wkEx93 (sma-10p::lambik)*	<0.001 a	97±3	49

**^a^**Compared to *sma-10(wk66)*.

The p value is the probability that the null hypothesis, that the mean body length of each mutant line is the same as the background body length, is true. “% WT” is the ratio of the indicated strain mean to the wild-type strain mean ±95% confidence interval. n, number of animals measured.

### SMA-10 Promotes BMP Signaling in Mammalian Cells

**Figure 3:**
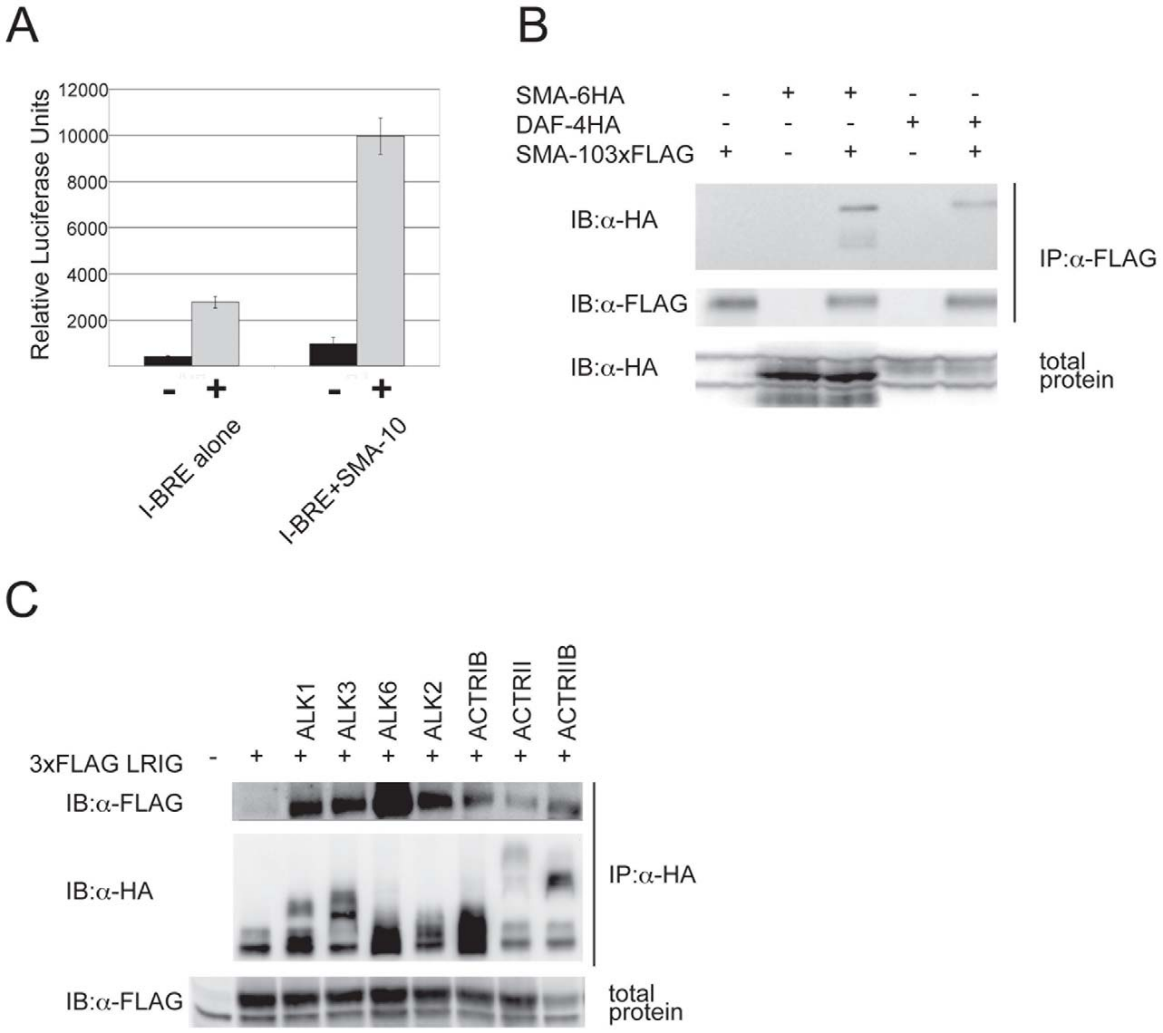
SMA-10 binds BMP receptors and enhances BMP signaling. SMA-10 enhances BMP signaling in HepG2 cells (A). Cells were transfected with a BMP-responsive transcriptional reporter (I-BRE:luciferase) and either SMA-10 or a control vector. Cells were either left unstimulated (black bars) or were stimulated with 2.5 nM BMP2 (grey bars). The average of four transfected wells is shown plus and minus the standard deviation. SMA-10 binds SMA-6 and DAF-4 (B). 293T cells were co-transfected with FLAG-tagged SMA-10 and HA-tagged SMA-6 or DAF-4 receptors. Lysates were immunoprecipitated with anti-FLAG (SMA-10) and blotted for HA (SMA-6 or DAF-4). LRIG1 is able to interact with several mammalian BMP receptors (C). Cells were co-transfected with various HA-tagged BMP receptors and 3x FLAG-tagged LRIG1, then lysates were immunoprecipitated with anti-HA antibody and blotted with anti-FLAG to detect LRIG1.

Our genetic data demonstrate that SMA-10 acts within a nematode BMP-like pathway to promote signaling. To test whether this function is conserved, we asked if SMA-10 could regulate BMP signaling in mammalian cells. We used a standard reporter assay in a BMP-responsive human cell line (HepG2), the BMP-response element from the Smad7 gene driving luciferase [39]. BMP2 induced reporter activity significantly over the controls, whereas in the presence of SMA-10, the response of the promoter was increased 4.2-fold over BMP2 alone (Figure 3A). Thus, SMA-10 can directly promote mammalian BMP signaling.

### SMA-10 Binds BMP Receptors SMA-6 and DAF-4

The genetic and molecular data strongly support a model in which SMA-10 acts on the extracellular surface of the plasma membrane, and could act via physical interactions with ligand, receptors, or ligand and receptors. To examine these possibilities and to provide mechanistic insights into SMA-10's function, we asked whether SMA-10 directly binds to ligand or to the DBL-1 receptors, SMA-6 and DAF-4. For the first experiment, we employed affinity labeling and substituted BMP2 for DBL-1, since DBL-1 protein is not readily available and BMP2 has been shown to physically interact with other DBL-1 pathway members [17], [24]. SMA-10 was FLAG-tagged and BMP2 ligand was radio-iodinated with ^125^I. After anti-FLAG immunoprecipitation and blotting, the blot was exposed to film. However, we failed to detect any binding of BMP2 to SMA-10 (Figure S1). We next asked whether SMA-10 interacted with the receptors. FLAG-tagged SMA-10 and HA-tagged receptors were transfected into 293T cells. To isolate SMA-10 and associated proteins from cells, lysates were first immunoprecipitated with anti-FLAG antibody and blotted. The blot was then probed with anti-HA antibody to determine whether receptors were bound to SMA-10. Under these conditions, both SMA-6 and DAF-4 receptors co-immunoprecipitated with SMA-10 (Figure 3B). Thus, SMA-10 physically interacts with receptors and not with the ligand.

### LRIG1 Binds Mammalian BMP Receptors

Given that SMA-10 binds the Sma/Mab pathway receptors, we tested whether this interaction was conserved in a mammalian system. Using the mammalian ortholog LRIG1 and mammalian BMP receptors, we assayed the ability of LRIG1 to bind the receptors. FLAG-tagged LRIG1 and HA-tagged versions of BMP receptors were transfected into mammalian cells. Lysates were immunoprecipitated with anti-FLAG antibody to pull down LRIG1, blotted, and probed with anti-HA antibody to determine whether LRIG1 binds any BMP receptors. We found that LRIG1 interacted strongly with type I receptor ALK6 and more weakly with the type I receptors ALK1, ALK2, ALK3, and ActRIB (Figure 3C). In addition, we detected weak interactions with type II receptors ActRII and ActRIIB (Figure 3C). Thus, LRIGs interact with BMP receptors and represent a new class of BMP receptor-associated proteins.

## Discussion

Here we describe a conserved, novel regulator of BMP signaling, SMA-10, which is absolutely required for body size signaling in *C. elegans*. Loss of SMA-10 function results in a phenotype similar to a loss of any of the Sma/Mab pathway components. Molecular and genetic analyses place its function at the level of the cell membrane between the ligand and the receptors. Several lines of evidence support a model in which SMA-10 and its orthologs are conserved regulators of BMP signaling. The *Drosophila* homolog *lambik* rescues *sma-10(lf)* animals. Consistent with genetic evidence that SMA-10 is a positive effector of BMP signaling, SMA-10 stimulates a positive BMP-specific response in human cells. Furthermore, SMA-10 and LRIG1 physically interact with BMP type I and type II receptors. LRIGs have previously been shown to regulate EGF and Met receptor tyrosine kinase signaling [40], [41], and recently the insect-specific *kekkon5* was shown to genetically interact with a BMP signaling pathway [29]. Our work reveals a new, conserved role for the LRIG subfamily in BMP receptor signaling.

### SMA-10 Acts in BMP Signaling in *C. elegans*

We propose that SMA-10 acts at the hypodermal membrane surface, where the Sma/Mab receptors are located, to facilitate receptor signaling. Our genetic evidence shows that the SMA-10 protein acts between the secreted ligand and the transmembrane receptors (Table 2). The structure of SMA-10 indicates that it contains a secretion signal and a transmembrane region (Figure 2). Subcellular localization of a rescuing translational SMA-10:GFP fusion shows SMA-10 in hypodermal tissues and at cell membrane surfaces (Figure 1C and 1E). The requirement for *sma-10* expression in the hypodermis to rescue the *sma-10(lf)* small body size further supports the model that SMA-10 acts at the hypodermal membrane to facilitate DBL-1 binding to its receptors (Table 3). We predict that the extracellular domain is responsible for this rescuing effect, as SMA-10's C-terminal intracellular tail is short (19 amino acids) and not conserved with Lambik, which has SMA-10 function, and SMA-10 is able to rescue when tagged with GFP at the C-terminus. We suggest that this intracellular sequence is not critical for transducing BMP signals.

Mutant animals for all previously characterized Sma/Mab core-signaling components have alterations in both body size and male tail defects. However, *sma-10* only affects body size. This can be explained if *sma-10* acts in a tissue-specific manner, namely the hypodermis, where DBL-1activated pathway signaling is required for body size control. Previous studies of targets downstream of this pathway (*lon-1*, *mab-21*, and *mab-23*) and one upstream regulator (LON-2) have shown that functions in the body size and male tail pathways are separable [22], [24], [30], [31], [42]. Given that sequencing reveals that one of the *sma-10* alleles (*wk88*) is a presumed null, we favor the model that SMA-10 acts in a tissue-specific manner to enhance signal strength in the cells contributing to body size but not to the male tail. Consistent with this reasoning, differences in the requirement of signal strength between the tail and the hypodermis are seen with a hypomorphic allele of *sma-6*, where body size is reduced while tail morphology is normal [18]. It remains to be seen whether differential responses to Sma/Mab pathway dosage differences are sufficient to explain this tissue specificity or if there exists another male-specific factor that functions, like SMA-10 in the hypodermis, to positively regulate Sma/Mab signaling in the male tail. We note that all the *sma-10* alleles result in small animals that are not as small as animals with loss-of-function mutations in previously identified core-signaling components. If tissue specificity in other organisms is a common feature, then this could explain why *sma-10* orthologs were not previously identified as BMP signaling modulators. We note that mammals have three LRIGs, and mutating a single LRIG (LRIG1, a.k.a. LIG-1) results in a mild hyperplasia of epidermal cells [43]. This is a seemingly minor defect, but loss of LRIG1 function may be partially compensated by the other two functional LRIGs. Therefore, redundancy may also explain why LRIGs have eluded identification as regulators of BMP signaling in higher organisms.

### SMA-10 Is a Conserved LRIG Family Member

The LRR and Ig-like domains exist singly in many other proteins, but only in the LRIG family do they exist in the same protein. The number of protein domains in the SMA-10/LRIG family, which excludes the insect Kekkon subfamily, is invariant. These domains have been shown to be involved with protein-protein interactions [44], [45]. In these studies, we show that SMA-10 and LRIG1 bind BMP type I and type II receptors, and our genetic evidence and studies based on overexpression in mammalian cells suggests that these proteins are positively required for BMP family signal transduction.

Kekkon 1 has been shown to be a negative regulator of the EGF pathway in *Drosophila* and acts by binding to the receptors [28]. Given the involvement of Kekkon 1 in EGF signaling, LRIG1 was tested for EGFR regulation in mammalian tissue culture [40]. LRIG1 was shown to also bind EGFRs and enhance their degradation [40]. Signaling of EGFs through EGFRs promotes cell proliferation. LRIG1 negatively regulates cell proliferation by down-regulating EGFR responsiveness by increasing activated receptor ubiquitination [28], [40]. LRIG1 binds the E3 ubiquitin ligase c-Cbl, bringing it into the EGFR complex. EGFR then phosphorylates c-Cbl, thereby activating it and promoting ubiquitination and degradation of both LRIG1 and EGFRs. LRIG1's amino acids 900 to 930 contain the binding site for c-Cbl [40], within its intracellular domain that is not shared by other LRIGs, SMA-10, or Lambik. An interaction of c-Cbl with LRIG2 or LRIG3 has not been demonstrated [46]. The ectodomain of LRIG1 alone inhibits EGFR signaling, but does so in a ubiquitin-independent fashion, showing that EGFR inhibition by LRIGs is not exclusively through ubiquitination [47]. EGFR signaling in *C. elegans*, mediated by a single EGFR (LET-23), directs several embryonic and larval cell fates and also ovulatory contractions in adult hermaphrodites [48]. We did not see any obvious defects associated with EGFR in *sma-10(lf)* animals that might suggest an interaction with EGFR. LRIG1 also binds to and inhibits signaling by hepatocyte growth factor receptor (Met), a tyrosine kinase that in many known cancers is mutated or misregulated to promote invasive growth, though its mechanism of inhibition is ubiquitin-independent [41]. *C. elegans* has no recognized Met receptor tyrosine kinase [49]. Another Kekkon, Kekkon5, affects BMP signaling, but the model, based on genetic and structure-function analyses, proposes that Kekkon5 regulates ligand distribution or activity rather than acts directly on receptors, as we show here for both *C. elegans* LRIG SMA-10 and mammalian LRIG1 [29].

Although their expression appears to be universal, human LRIGs are differentially expressed in various cancer cell types, being down regulated in many types studied, but being upregulated in others [46]. Various explanations have been proposed based on the current understanding of LRIG function [46]. Our research showing LRIG interaction with BMP receptors (BMPRs) leads us to propose a new model, where the cell-specific levels of BMP and EGF activity, which negatively and positively regulate cell growth, respectively, determine the cell's response to LRIG exposure.

The observation that SMA-10 localizes to intracellular puncta (Figure 1C) is reminiscent of endocytic vesicles, and suggests a model of action for SMA-10 and the Sma/Mab signaling pathway [50]. In other systems, there is evidence that TGF-β superfamily pathway signaling can be activated via receptor monoubiquitination and receptor complex endocytosis into early vesicles (reviewed in [51], [52]). SMA-10 may thus promote signaling by facilitating receptor internalization into early endosomes.

This work identifies a new, conserved component of BMP signaling, SMA-10/LRIG. Mammalian members play known roles in some receptor tyrosine kinase pathways, and this work identifies a new role for this family in BMP receptor serine/threonine kinase signaling. We have shown that two members of this family physically interact with both the type I and type II BMP receptors, and *C. elegans* SMA-10 enhances signaling in both the nematode and in mammalian cells.

## Materials and Methods

### Strains

Animals were maintained according to standard protocols [26]. All mutant strains used in this study were derived from the wild-type Bristol strain N2, and some mapping was accomplished using the wild-type Hawaii isolate CB4856. Alleles used include *sma-10(wk26, wk66, wk88, wk89, wk90)* [25], *lon-2(e678)*, *sma-6(wk7)*, *ctIs40* [ZC421 (*dbl-1(+)*) + pTG96 (*sur-5::gfp*)] [23], *bxIs16* [*tph1::gfp* + *cat-2::yfp*] [42]; and *nIs128* [*pkd-2::gfp*] [53]. Arrays made for this study are *wkEx47* (*sma-10p::sma-10:gfp* + pUC18 filler DNA, 50 ng/µl each), *wkEx91 (myo-2p::sma-10(+))*, *wkEx92 (rol-6p::sma-10(+))*, *wkEx93 (sma-10p::lambik)*, *texEx190 (sma-6p::sma-6(+):gfp)*, and *texEx195 (sma-10p::sma-10(yk352c5)*.

Creation of transgenic arrays was performed by standard microinjection techniques [54]. Genomic *sma-10* or *Drosophila lambik* cDNA was cloned into nematode expression vector pPD95.75 with the appropriate promoter sequence. *wkEx47* was made by removing the stop codon of the *sma-10* genomic sequence and fusing the *gfp* sequence in-frame at the 3′ end. Transgenic animals were generated by germline microinjection, using constructs at 50 ng/µl (HW480 *sma-10p::lambik*, CZ10.2 *sma-10p::sma-10:gfp*, and CZ9.1 *sma-10(yk352c5*)) or 0.5 ng/µl (HW469 *rol-6p::sma-10* and HW477 *myo-2p::sma-10*) with the co-injection marker *ttx-3p::rfp* or *ttx-3p::gfp* at 50 ng/µl (with HW480 and CZ9.1) or 100 ng/µl (with HW469 and HW477). *ttx-3p* drives expression in AIY interneurons. One representative stable line for each transgene was measured.

### Isolation of *sma-10* Mutant Animals

Isolation of *sma-10(wk26)* and *sma-10(wk66)* was previously described [25]. In an effort to identify additional alleles of genes that act BMP signaling, *lon-2(e678)* hermaphrodites were mutagenized with 50 mM ethyl methanesulfonate (EMS) using standard procedures [26]. Mutagenized P_0_ animals were transferred to plates and allowed to segregate self-progeny. F_1_ animals were transferred to new plates to segregate progeny, which were then scored for a small phenotype in a quarter of the population. From about 9,000 mutagenized genomes screened, three additional alleles of *sma-10* were isolated, *wk88*, *wk89*, and *wk90*. These alleles, as well as the two alleles isolated in the Sma screen [25], were outcrossed five times before further analyses were done.

### Phenotypic Analyses

To measure body size, animals were picked at the L4 stage and photographed as young adults about 24 hours later. Images from individual animals were captured from a dissecting microscope using an Optronics MagnaFire CCD camera system and software (Optronics, Goleta, CA). Perimeters (Table 1) or lengths (Table 2, Table 3, Table 4) of animals were determined by using Image-Pro Plus measurement software (Media Cybernetics, Inc., Silver Spring, MD). The images for Figure 1C and 1D were captured using an Axiovert 200 M microscope (Carl Zeiss MicroImaging, Oberkochen, Germany) equipped with a digital CCD camera (C4742-95-12ER, Hamamatsu Photonics, Hamamatsu, Japan) and were deconvolved with AutoDeblur software (AutoQuant Imaging, Watervliet, NY). The Figure 1E image was obtained on an Olympus IX81 with a Carv Nipkow disk confocal unit (Atto Biosciences, Rockville, MD) and SensiCam QE camera (Cooke Corp., Auburn Hills, MI).

We performed statistical analyses on these measurements. Individual measurements from each strain were averaged. We determined the ratio and 95% confidence interval of the average measurement (mean) of each strain to the wild-type strain mean. To verify the significance of our findings, we tested the null hypothesis that the ratios of the compared means are the same. The ratios compared were the double mutant strain mean/wild type mean to the single *sma*/transgenic mutant strain mean/wild type strain mean. For populations measured on the same day, the denominator (the average of wild-type measurements) was the same and a Student's t-test was used to test the null hypothesis. For populations measured on different days, the Welch-Satterthwaite equation was used to calculate the effective degrees of freedom for this 2-tailed t-test for two ratios. We determined the value of t with the calculated degrees of freedom and compared the t-value to the Student's t table to construct the probability (p) of the null hypothesis.

### Epistasis Analyses

*sma-10(wk66); lon* double mutant animals were constructed by crossing heterozygous *sma-10(wk66)* males with *lon-2(e678)* or *lon-1(wk50)* hermaphrodites, with animals overexpressing an integrated transgene with wild-type *dbl-1 (ctIs40)*, or with animals overexpressing an extrachromosomal array encoding functional SMA-6 (*texEx190*) [15]. Wild-type F_1_ were isolated and small and long F_2_ animals were picked to individual plates. The F_3_ generation was then examined for the presence of long animals from a small F_2_ parent or small animals from a long F_2_ parent.

### Positional Cloning of *sma-10*

*sma-10(wk66)* was previously mapped to chromosome IV by two-factor crosses [25]. We further refined its position by standard three-factor mapping and single nucleotide polymorphism mapping [26], [55]. We used microinjection and germline transformation rescue [54] to discover that YAC Y80C9 and cosmid T21D12 rescue the small phenotype of *sma-10(wk66)* animals. Each predicted gene on T21D12, with at least 1.5 kb of promoter sequence, was amplified by the polymerase chain reaction (PCR). The DNA product was purified and injected into *sma-10(wk66)* animals. Only T21D12.9 rescued the small phenotype. PCR confirmed altered or deleted sequences in all five *sma-10* alleles.

### Cell Culture

293T cells were cultured in Dulbecco's modified Eagle's medium containing high glucose and supplemented with 10% fetal bovine serum (FBS). HepG2 cells were cultured in minimal essential media supplemented with 1% non-essential amino acids and 10% FBS. Cells were transfected by calcium phosphate and lysed 48 hours after transfection in TNTE buffer containing 0.5% Triton-X-100 (150 mM NaCl, 50 mM Tris ph 7.4, and 1 mM EDTA) [56]. Immunoprecipitations were carried out using M2 anti-FLAG (Sigma) or 12CA5 anti-HA (made in-house) followed by incubation with Protein-G Sepharose beads (Amersham Biosciences, Uppsala, Sweden). Immunoprecipitates were then washed four times with lysis buffer containing 0.1% Triton-X-100. Proteins were separated by SDS-PAGE and immunoblotted with anti-HA (12CA5) or anti-FLAG (M2).

### Signaling Assays

Transcriptional assays were carried out using a previously described BMP-responsive element from the mouse Smad7 gene (I-BRE) driving firefly luciferase [39]. Luciferase assays were carried out as described [39]. *Renilla* luciferase expressed from the CMV promoter was used as an internal control for transfection efficiency. Firefly luciferase values were normalized using *Renilla* luciferase values.

### Constructs

1.2 kb promoter sequence 5′ of the *sma-10* open reading frame driving expression of genomic *sma-10* was sufficient to rescue body size defects in *sma-10(lf)* animals, and this same promoter region was fused to *Drosophila lambik*/CG8434 cDNA to address conservation of LRIG family function. Tissue specific expression was determined by driving expression of *sma-10* genomic sequence with *myo-2* (pharyngeal expression) or *rol-6* (hypodermal expression) promoters [57]. We generated GFP-tagged SMA-10 by fusing the eGFP sequence to the 3′ terminus of wild-type *sma-10* genomic sequence that lacked its termination codon.

The rescuing *sma-6* construct was made by fusing the mCherry sequence in frame to the 3′ end of genomic *sma-6* and expressing it using 1260 bp *sma-6* promoter sequence.

3x FLAG SMA-10 was made by fusing the full-length *sma-10* cDNA from the first Sal I site in frame to the 3x FLAG signal peptide containing vector p3xFLAG-CMV8 (Sigma). Similarly, we constructed the 3x FLAG-tagged LRIG-1 by subcloning the LRIG cDNA from an endogenous Sal I restriction site to the C-terminus into p3xFLAG-CMV8. This construct encodes a protein with the 3x FLAG tag between the N-terminal signal sequence and the first LRR of LRIG1. SMA-6 was C-terminally tagged with the HA1 epitope of influenza virus hemagglutinin (HA) and was cloned into the mammalian expression vector pCMV5 (T. Reguly and J. Wrana, unpublished work). DAF-4DC-HA is HA-tagged DAF-4 with a deletion of its C-terminal tail (T. Reguly and J. Wrana, unpublished work). ALKIHA, ALK3HA, ALK6HA, ALK2HA, ActRIBHA, ActRIIHA, ActRIIBHA have been previously described [58]–.

## Supporting Information

Figure S1 SMA-10 does not bind BMP ligands. HepG2 cells were transfected with either HA-tagged DAF-4, the control vector, or HA-tagged SMA-10 and incubated with 0.5 nM ^125I^-BMP2. Lysates were collected and immunoprecipitated with HA antibody. Samples were split and separated on SDS-PAGE gels and scanned for visualization of ^125I^-BMP2 or immunoblotted with HA antibody for visualization of DAF-4 or SMA-10. DAF-4 binds ^125I^-BMP2, while neither SMA-10 nor the vector bind ^125I^-BMP2. The bottom panel shows that both DAF-4 and SMA-10 were present in the cell lysates.
(4.94 MB TIF)

Table S1 *sma-10* cDNA rescues Body Size *sma-10(lf)* Mutants. Body lengths of staged one-day adult hermaphrodites were measured. *sma-10(wk66)* animals were non-transgenic siblings of *sma-10(wk66); texEx195* animals. The p value is the probability that the null hypothesis, that the mean body length of the transgenic line is the same as the *sma-10(wk66)* mean body length, is true. “% *sma-10(wk66)*” is the ratio of the transgenic strain mean to the non-transgenic *sma-10(wk66)* strain mean ±95% confidence interval. n, number of animals measured.
(0.03 MB DOC)
