# Supplementary figures and images for: Caenorhabditis elegans SMA-10/LRIG Is a Conserved Transmembrane Protein that Enhances Bone Morphogenetic Protein Signaling
Source: PLoS Genet. 2010 May 20;6(5):e1000963. doi: 10.1371/journal.pgen.1000963 (PMC2873917; doi:10.1371/journal.pgen.1000963)

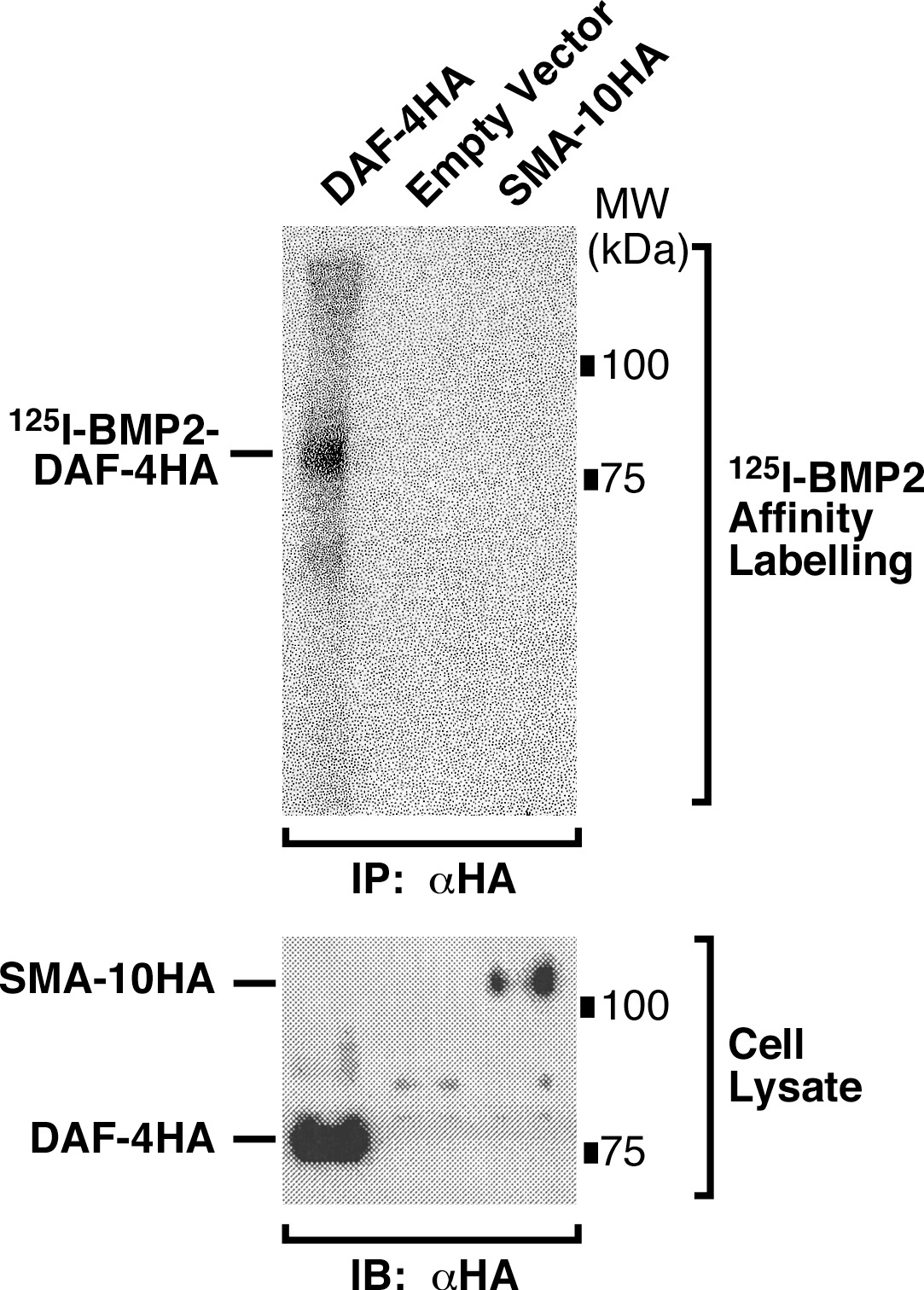

Supplement: Figure S1 — SMA-10 does not bind BMP ligands. HepG2 cells were transfected with either HA-tagged DAF-4, the control vector, or HA-tagged SMA-10 and incubated with 0.5 nM 125I-BMP2. Lysates were collected and immunoprecipitated with HA antibody. Samples were split and separated on SDS-PAGE gels and scanned for visualization of 125I-BMP2 or immunoblotted with HA antibody for visualization of DAF-4 or SMA-10. DAF-4 binds 125I-BMP2, while neither SMA-10 nor the vector bind 125I-BMP2. The bottom panel shows that both DAF-4 and SMA-10 were present in the cell lysates. (4.94 MB TIF) [file pgen.1000963.s001.tif]
