# Supplementary material for: Caenorhabditis elegans SMA-10/LRIG Is a Conserved Transmembrane Protein that Enhances Bone Morphogenetic Protein Signaling
Source: PLoS Genet. 2010 May 20;6(5):e1000963. doi: 10.1371/journal.pgen.1000963 (PMC2873917; doi:10.1371/journal.pgen.1000963)
Supplement: Table S1 — sma-10 cDNA rescues Body Size sma-10(lf) Mutants. Body lengths of staged one-day adult hermaphrodites were measured. sma-10(wk66) animals were non-transgenic siblings of sma-10(wk66); texEx195 animals. The p value is the probability that the null hypothesis, that the mean body length of the transgenic line is the same as the sma-10(wk66) mean body length, is true. “% sma-10(wk66)” is the ratio of the transgenic strain mean to the non-transgenic sma-10(wk66) strain mean ±95% confidence interval. n, number of animals measured. (0.03 MB DOC) [file pgen.1000963.s002.doc]

| genotype | p value | % *sma-10(wk66)* | n |
| --- | --- | --- | --- |
|  |  |  |  |
| *sma-10(wk66)* |  | 100±7 | 18 |
| *sma-10(wk66); texEx195 (sma-10p::yk352c5)* | p < 0.001 | 126±6 | 28 |
